# Role of Magnetic Resonance Spectroscopy in Evaluating Choline Levels in Gallbladder Carcinoma: A Comprehensive Review

**DOI:** 10.7759/cureus.66205

**Published:** 2024-08-05

**Authors:** Anjali Kumari, Gaurav Mishra, Pratapsingh Parihar, Sakshi S Dudhe

**Affiliations:** 1 Radiodiagnosis, Jawaharlal Nehru Medical College, Datta Meghe Institute of Higher Education and Research, Wardha, IND

**Keywords:** cancer staging, diagnostic imaging, biomarkers, choline metabolism, magnetic resonance spectroscopy (mrs), gallbladder carcinoma

## Abstract

Gallbladder carcinoma (GBC) presents a significant clinical challenge due to its aggressive nature and often asymptomatic progression, resulting in late-stage diagnoses and a poor prognosis. Early detection and accurate staging are pivotal for improving patient outcomes, highlighting the critical role of advanced imaging techniques in oncological practice. Magnetic resonance spectroscopy (MRS) has emerged as a valuable non-invasive tool capable of assessing biochemical changes within tissues, including alterations in choline metabolism-a biomarker indicative of cell membrane turnover and proliferation. This review explores the application of MRS in evaluating choline levels in gallbladder carcinoma, synthesizing current literature to elucidate its potential in clinical settings. By analyzing studies investigating the correlation between choline levels detected via MRS and tumor characteristics, this review underscores MRS's role in enhancing diagnostic precision and guiding therapeutic decision-making. Moreover, it discusses the challenges and limitations associated with MRS in clinical practice alongside future research and technological advancement directions. Ultimately, integrating MRS into the diagnostic armamentarium for gallbladder carcinoma promises to improve early detection and treatment outcomes. This review provides insights into the evolving landscape of MRS in oncology, emphasizing its contribution to personalized medicine approaches aimed at optimizing patient care and management strategies for GBC.

## Introduction and background

Gallbladder carcinoma (GBC) represents a formidable challenge in oncology, characterized by its aggressive nature and often asymptomatic progression until advanced stages. As the sixth most common gastrointestinal malignancy worldwide, GBC poses significant diagnostic and therapeutic challenges due to its insidious onset and propensity for late-stage detection [1]. The lack of early symptoms often results in diagnosis at a stage where cancer has already advanced, making treatment more complex and decreasing the likelihood of favorable outcomes. Therefore, early identification of GBC is crucial for improving patient prognosis, underscoring the critical importance of precise staging and timely intervention [2].

Imaging techniques are essential in the comprehensive management of cancer, serving as crucial tools for the initial detection, accurate staging, and monitoring of treatment response [3]. Among these techniques, magnetic resonance spectroscopy (MRS) has emerged as a promising modality for evaluating biochemical alterations associated with cancerous tissues. MRS allows for the non-invasive assessment of metabolite levels within tissues, providing insights into the cellular processes often altered in malignancies such as GBC. This technique is particularly valuable as it offers detailed biochemical information that complements traditional imaging methods, potentially leading to more accurate diagnoses and a better understanding of tumor biology [3,4].

This review aims to explore the role of MRS in evaluating choline levels, which is a key biomarker of cellular membrane turnover and proliferation, in the context of gallbladder carcinoma. Choline is involved in synthesizing phosphatidylcholine (PC), an essential component of cell membranes, and its elevated levels are often indicative of increased cell membrane turnover, a common feature in cancerous tissues. By examining current literature and clinical applications, this review seeks to highlight the potential of MRS as an adjunctive imaging modality. The goal is to enhance diagnostic accuracy and guide therapeutic strategies in GBC, offering a more nuanced approach to managing this challenging malignancy.

## Review

Literature search strategy

This comprehensive review was conducted through an extensive literature search to gather relevant studies on the role of magnetic resonance spectroscopy (MRS) in evaluating choline levels in gallbladder carcinoma (GBC). The primary databases used for the literature search included PubMed, MEDLINE, EMBASE, Scopus, and Google Scholar, selected for their extensive medical and scientific literature coverage. The following search engines and databases were utilized: PubMed, MEDLINE, EMBASE, Scopus, and Google Scholar. The search terms and keywords used in the search strategy included: "Magnetic Resonance Spectroscopy," "Choline," "Gallbladder Carcinoma," "Gallbladder Cancer," "Choline Metabolism," "MRS," "Diagnostic Imaging," "Oncology," "Cancer Biomarkers," and "Non-invasive Imaging." The keywords were combined using Boolean operators (AND, OR) to ensure a comprehensive search, such as "Magnetic Resonance Spectroscopy AND Gallbladder Carcinoma," "Choline AND Gallbladder Cancer," and "MRS AND Choline Metabolism AND Oncology."

The inclusion criteria encompassed studies that discussed the application of MRS in oncology, specifically related to choline metabolism; research articles focusing on gallbladder carcinoma and its diagnosis using imaging techniques; clinical trials, reviews, and meta-analyses that evaluated the efficacy of MRS in detecting choline levels in cancer tissues; and articles published in English. Exclusion criteria included studies not involving humans or not specifically addressing gallbladder carcinoma, articles that did not provide clear data or outcomes related to the use of MRS, and publications in languages other than English. A team of researchers, Anjali Kumari, Gaurav Mishra, Pratapsingh Parihar, and Sakshi S. Dudhe, performed the literature search. Each team member independently conducted the initial search to ensure comprehensive topic coverage. The initial search results were then compiled, and duplicate entries were removed. The titles and abstracts of the identified articles were screened to determine their relevance to the review topic. Full-text articles of potentially relevant studies were retrieved and evaluated against the inclusion and exclusion criteria. Data extraction was carried out systematically, focusing on study design, patient population, MRS methodology, choline measurement, outcomes, and key findings related to the use of MRS in diagnosing and managing GBC.

Fundamentals of magnetic resonance spectroscopy (MRS)

Explanation of MRS as a Non-Invasive Imaging Modality

Magnetic resonance spectroscopy (MRS) is a non-invasive imaging technique that provides valuable insights into the biochemical composition and metabolic activity of tissues, complementing the anatomical details obtained from magnetic resonance imaging (MRI) [5]. MRS operates on detecting the resonance frequencies of atomic nuclei such as hydrogen-1, carbon-13, and phosphorus-31 when exposed to a strong magnetic field. These resonant frequencies are measured and presented as a spectrum, which reveals the relative concentrations of various metabolites within the tissue [5]. MRS has been utilized to study metabolic changes in numerous brain diseases, including tumors, stroke, epilepsy, and Alzheimer's disease. Additionally, it has been applied to investigate metabolism in other organs, such as the heart, liver, and muscles. MRS can provide crucial information about the malignancy level of brain tumors, the extent of neuronal damage in head trauma, and the presence of infections like brain abscesses [6]. Technically, MRS is typically conducted as an adjunct to a standard MRI exam using the same MRI scanner hardware. Higher magnetic field strengths (e.g., 3T or 7T) offer improved signal-to-noise ratios and better separation of metabolite peaks than lower field strengths like 1.5T. Unlike MRI, which images a larger field of view, MRS acquires signals from a single localized region, known as a voxel [7].

Principles of MRS in Detecting Biochemical Changes

Magnetic resonance spectroscopy (MRS) can non-invasively measure the relative concentrations of various metabolites within tissues, such as the brain, liver, and other organs. When placed in a strong magnetic field, it detects the resonance frequencies of atomic nuclei like hydrogen-1, carbon-13, and phosphorus-31. These resonant frequencies are displayed as a spectrum that reveals the relative amounts of different metabolites [5]. Abnormal levels of certain metabolites, such as elevated choline or decreased N-acetyl aspartate (NAA), can indicate pathological conditions like tumors, infections, demyelination, and more. The metabolite profile detected by MRS can help differentiate between various disease states and guide diagnosis and treatment monitoring [8]. MRS provides functional, biochemical information that complements the anatomical details obtained from magnetic resonance imaging (MRI). MRI and MRS can enhance diagnostic accuracy and prognostic value for conditions such as brain tumors by assessing structural and metabolic changes [9].

Specific Focus on Choline as a Biomarker

The role of magnetic resonance spectroscopy (MRS) in evaluating choline levels in gallbladder carcinoma (GBC) is an important area of research. MRS enables the non-invasive assessment of metabolite levels within tissues, including choline, a component of cell membranes. Elevated choline levels are associated with increased cell proliferation and malignant transformation, making MRS a potential tool for detecting increased choline levels as a biomarker for GBC detection [10]. Magnetic resonance imaging (MRI) provides high-resolution gallbladder imaging, allowing for a detailed assessment of tumor size, local invasion, and vascular involvement. MRS can complement MRI by measuring choline levels within tumor tissue, potentially indicating malignancy. Previous studies have demonstrated that MRS can help distinguish malignant tumors from benign lesions based on elevated choline levels. Combining MRI and MRS can enhance diagnostic accuracy for GBC [11]. MRS findings of elevated choline levels in GBC can be correlated with histopathological findings to better understand the role of choline as a biomarker for this aggressive malignancy. This can provide insights into the relationship between choline metabolism and underlying tumor biology. Understanding these metabolic changes could lead to developing targeted therapies and personalized treatment approaches for GBC patients [12]. The feasibility and safety of performing MRI and MRS in patients with GBC will also be assessed to ensure the clinical applicability of these imaging techniques in managing GBC. If proven feasible and safe, MRS could become a valuable tool for early detection, monitoring treatment response, and guiding personalized therapy in GBC patients [13].

Choline metabolism in cancer

Role of Choline in Cell Membrane Synthesis and Turnover

**Figure 1:**
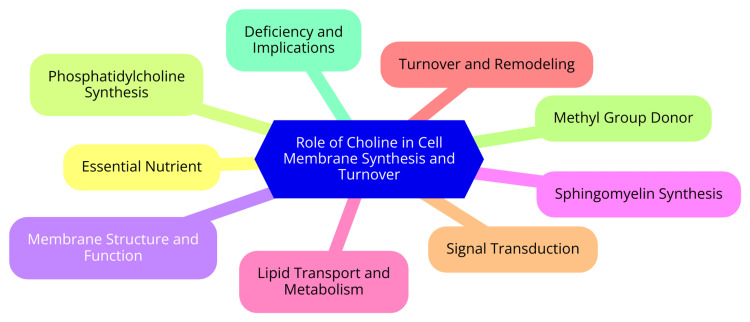
The role of choline in cell membrane synthesis and turnover. Image Credit: Dr Anjali Kumari.

The role of choline in cell membrane synthesis and turnover is multifaceted. Choline is an essential nutrient that serves as a precursor for synthesizing phosphatidylcholine (PC), the most abundant phospholipid in cell membranes. This makes choline a crucial component for the continuous synthesis and turnover of phospholipids, which are the structural building blocks of cell membranes [14]. The choline transport into cells is facilitated by specialized choline transporter proteins, such as the choline transporter-like protein 1 (CTL1/SLC44A1). This active transport of choline is essential for maintaining membrane integrity and function, as it provides the necessary raw materials for the dynamic nature of cell membranes [15]. The regulation of choline metabolism and transport is closely linked to the dynamic properties of cell membranes. Factors affecting choline availability, such as dietary intake, can influence cell membranes' composition and fluidity, impacting cellular processes like signaling, proliferation, and apoptosis [16]. Dysregulation of choline metabolism and transport has been implicated in the development and progression of certain cancers and neurological disorders. As detected by magnetic resonance spectroscopy (MRS), elevated choline levels can serve as a biomarker for malignant transformation and tumor aggressiveness. This underscores the importance of understanding the role of choline in cell membrane synthesis and turnover [17]. The role of choline in cell membrane synthesis and turnover is shown in Figure 1.

Altered Choline Metabolism in Cancer Cells

Cancer cells exhibit a distinct metabolic phenotype characterized by elevated levels of choline-containing metabolites, such as phosphocholine (PCho) and total choline (tCho), compared to normal cells. This "cholinic phenotype" can be detected non-invasively using magnetic resonance spectroscopy (MRS) techniques [18]. The increased choline metabolites in cancer cells are driven by alterations in the activity of enzymes involved in choline metabolism, including increased expression and activity of choline kinase-alpha (ChoK-α), which converts choline to phosphocholine, as well as changes in choline transporters and other enzymes like phospholipases C and D [19]. Oncogenic signaling pathways, such as rat sarcoma (RAS) and PI3K-AKT, and transcription factors, such as HIF-1, regulate aberrant choline metabolism in cancer cells. There is a reciprocal interaction between choline metabolism and oncogenic signaling, where metabolic changes can also influence the activation of these signaling cascades [20]. Monitoring choline metabolite levels using MRS has significant diagnostic and therapeutic implications. Choline metabolite levels can provide a non-invasive method to assess tumor response to conventional chemotherapies and targeted anticancer drugs. Additionally, targeting enzymes involved in choline metabolism, such as ChoK-α, is being explored as a novel therapeutic approach for cancer treatment [21].

Potential Implications for Diagnostic Imaging

Early detection and accurate staging of gallbladder carcinoma (GBC) are crucial for improving patient outcomes, as early detection is essential for surgical resection, the primary curative treatment option. MRI and MRS offer non-invasive tools for this purpose. Elevated choline levels detected by MRS can indicate malignancy, aiding in the early detection of GBC [22]. Choline levels measured by MRS can serve as imaging biomarkers for GBC. Elevated choline levels are associated with increased cell proliferation and malignant transformation, making them potential biomarkers for early tumor growth detection and monitoring. Correlating MRS findings with histopathological results can provide insights into the relationship between choline metabolism and tumor biology, further validating the use of choline as a biomarker and improving the understanding of GBC pathogenesis and prognosis [23]. The non-invasive nature of MRI and MRS can reduce the need for invasive procedures such as biopsy or exploratory surgery, minimizing patient discomfort and reducing the risk of complications associated with invasive methods. Quantifying choline levels can also aid in monitoring treatment response and prognostication, leading to more personalized and effective patient management strategies [24]. However, the study protocol acknowledges the need for larger cohorts to validate findings and establish robust diagnostic and prognostic models. Additionally, standardizing imaging protocols and training healthcare professionals in these techniques will be crucial for the widespread implementation of this approach [25].

Clinical applications of MRS in gallbladder carcinoma

Review of Studies Using MRS to Evaluate Choline Levels in GBC

Magnetic resonance spectroscopy (MRS) has shown promise in evaluating choline levels as a gallbladder carcinoma (GBC) biomarker. Recent studies have investigated the role of MRS in assessing choline levels in GBC to improve early detection and guide personalized treatment strategies [26]. MRS can detect elevated choline levels within tumor tissue, which may indicate malignancy. High choline levels are typically observed in brain tumors due to increased membrane turnover. In gliomas, choline is elevated beyond the margins of contrast enhancement, indicating cellular infiltration. Similarly, MRS can potentially detect elevated choline levels as a biomarker for early GBC detection. However, distinguishing between radiation changes and tumor recurrence can be challenging, as recurrent tumors will have elevated choline levels, while radiation changes will show low NAA, choline, and creatine levels [27]. MRS findings in GBC may correlate with histopathological results. Although tumors usually exhibit high choline levels, low-grade malignant tumors may show a negative choline resonance peak at 3.22 ppm. In conclusion, MRS can play a significant role in evaluating choline levels as a biomarker for GBC. Further research is needed to validate its utility and establish its role in the early detection and management of this aggressive malignancy [28].

Comparison With Other Imaging Modalities

Magnetic resonance imaging (MRI) and magnetic resonance spectroscopy (MRS) have shown promise in evaluating gallbladder carcinoma (GBC) compared to other imaging modalities. Ultrasound is often the initial imaging modality used to evaluate the gallbladder, but it has limitations in precisely characterizing tumor extent and aggressiveness in GBC. Computed tomography (CT) can provide detailed information about tumor size, local invasion, and vascular involvement in GBC, but it also has limitations in differentiating malignant from benign gallbladder lesions [29]. MRI provides high-resolution gallbladder imaging, allowing for a detailed assessment of tumor characteristics. It can accurately assess GBC's size, resectability, and vascular involvement. MRS enables the non-invasive assessment of metabolite levels within tissues, offering insights into cellular metabolism and potential biomarkers for cancer detection and characterization. Specifically, MRS can detect elevated choline levels as a biomarker for early GBC detection [30]. MRS findings may correlate with histopathological findings in GBC, although low-grade tumors may show a negative choline peak. While conventional imaging modalities like ultrasound and CT have limitations in precisely characterizing GBC, MRI and MRS offer advanced techniques that can provide more detailed information about tumor characteristics and metabolic biomarkers. The combination of MRI and MRS shows promise in improving early detection and guiding personalized treatment strategies for this aggressive malignancy [8].

Challenges and Limitations of MRS in Clinical Practice

Magnetic resonance spectroscopy (MRS) has shown promise in various clinical applications, but it faces several challenges and limitations that hinder its widespread adoption in clinical practice. One of the primary challenges is the validation of MRS findings. Although MRS has been utilized in small studies to demonstrate its potential, translating these findings into clinical practice requires close collaboration between scientists, statisticians, and clinicians. This collaboration is essential for testing MRS against current "gold standards" and demonstrating its clinical utility [31]. Technical limitations also pose significant challenges to the clinical use of MRS. Detection and identification of low-concentration metabolites in vivo can be less effective than in vitro MRS and mass spectrometry techniques. Additionally, MRS faces technical challenges such as magnetic field inhomogeneity, chemical shift artifacts, and low signal-to-noise ratios, all of which require optimization. These technical limitations can affect the accuracy and reliability of MRS results, complicating its use in clinical decision-making [32]. Another challenge is establishing the clinical usefulness of MRS. While MRS has shown promise in applications such as brain tumor characterization and metabolic disorder diagnosis, its role in clinical trials and routine patient management still needs to be proven. The clinical usefulness of MRS has yet to be fully substantiated, and as access to MRS increases, appropriate evaluations of its strengths and weaknesses will be necessary. Clinicians must weigh the benefits of using MRS against potential drawbacks, such as the need for specialized equipment and the risk of false positives or negatives. Ultimately, the success of MRS in clinical practice will depend on its ability to demonstrate clear clinical benefits and improve patient outcomes [33].

Case studies and clinical outcomes

Presentation of Case Studies Demonstrating MRS Findings in GBC

Magnetic resonance spectroscopy (MRS) has shown promise in enhancing diagnostic accuracy and prognostic value for various cancers, including gallbladder carcinoma (GBC). MRS enables the non-invasive assessment of metabolite levels within tissues, offering insights into cellular metabolism and providing potential biomarkers for cancer detection and characterization [9]. One metabolite of particular interest is choline, a component of cell membranes whose elevated levels have been associated with increased cell proliferation and malignant transformation. Recent studies have investigated the role of MRI and MRS in evaluating choline levels in GBC to improve early detection and guide personalized treatment strategies for this aggressive malignancy [20]. A prospective observational study evaluated the role of MRI and MRS in 25 patients with suspected or confirmed GBC. The study found that elevated choline levels detected by MRS were associated with increased cell proliferation and malignant transformation in GBC tumors. Choline levels correlated with histopathological findings, suggesting its potential as a non-invasive biomarker for GBC detection and characterization [34]. Another case report described the MRS findings in a 62-year-old patient with GBC. MRS revealed significantly elevated choline levels within the tumor, which correlated with the histopathological diagnosis of high-grade adenocarcinoma. The elevated choline levels detected by MRS helped guide the treatment approach and provided prognostic information for this patient [8]. A retrospective analysis examined the utility of MRI and MRS in differentiating benign from malignant gallbladder lesions. The study included 20 patients with confirmed GBC and 15 patients with benign gallbladder diseases. MRS demonstrated significantly higher choline levels in the GBC group than in the benign group, with a sensitivity of 90% and specificity of 87% for detecting malignancy. These findings highlight the potential of MRS as a non-invasive tool for early GBC detection [35].

Correlation Between Choline Levels and Tumor Characteristics

Magnetic resonance spectroscopy (MRS) is promising in enhancing diagnostic accuracy and prognostic value for various cancers, including gallbladder carcinoma (GBC). This technique allows for the non-invasive assessment of metabolite levels within tissues, offering insights into cellular metabolism and providing potential biomarkers for cancer detection and characterization. A key metabolite of interest is choline, a component of cell membranes whose elevated levels have been linked to increased cell proliferation and malignant transformation [36]. Recent studies have explored the role of MRI and MRS in evaluating choline levels in GBC to improve early detection and guide personalized treatment strategies for this aggressive malignancy. One prospective observational study aimed to assess the spectrum of MRI findings, determine choline levels using MRS, evaluate the diagnostic accuracy of MRI, explore the potential of MRS in detecting choline levels as a biomarker, and investigate any correlation between choline levels and histopathological findings in GBC [9]. Elevated choline levels detected by MRS have been associated with increased cell proliferation and malignant transformation in various cancers, including GBC. A notable study found a strong linear correlation between choline levels and the Ki-67 proliferation index in homogeneous gliomas. This suggests that choline can be a reliable predictor of proliferative activity when the tumor appears homogeneous on MRI. However, no such correlation was observed in heterogeneous gliomas, underscoring the importance of tumor morphology in interpreting choline levels [37]. Choline levels can be influenced by tumor composition, with higher levels found in regions of active tumor compared to lower levels in areas of necrosis or edema. The median choline level in a tumor region may vary based on the relative proportions of tumor, necrosis, and edema, leading to variability in choline values. Quantifying choline levels in GBC tumors using MRS could aid in early detection, monitoring treatment response, and prognostication, thereby supporting more personalized patient management [8].

Impact on Treatment Planning and Patient Outcomes

Magnetic resonance spectroscopy (MRS) has the potential to significantly impact treatment planning and patient outcomes in gallbladder carcinoma (GBC). By non-invasively assessing choline levels within GBC tumors, MRS may provide a valuable biomarker for tumor aggressiveness and help guide personalized treatment strategies. Elevated choline levels detected by MRS have been linked to increased cell proliferation and malignant transformation in GBC, highlighting its potential clinical relevance. Quantifying choline levels using MRS could also assist in monitoring treatment response, enabling clinicians to evaluate the efficacy of therapies better and make informed decisions about continuing or modifying treatment [10]. Early detection and accurate staging of GBC are crucial for improving patient outcomes, as surgical resection remains the primary curative treatment option. MRI and MRS have shown promise in enhancing diagnostic accuracy and prognostic value for GBC by non-invasively assessing metabolite levels within tumors. Correlating choline levels measured by MRS with tumor characteristics and clinical outcomes could provide valuable insights into the role of choline in GBC pathogenesis and prognosis. If validated, MRS could become a non-invasive tool for early detection, staging, and treatment response monitoring in GBC, ultimately improving patient management and outcomes [38]. However, the current evidence is based on limited case studies, and further research is needed to validate these findings and establish robust diagnostic and prognostic models using MRS in GBC. Larger studies are necessary to confirm choline's utility as a reliable GBC biomarker. Additionally, widespread implementation of these techniques requires standardization of imaging protocols and training healthcare professionals to ensure accurate and reproducible results [39].

Future directions and research

Emerging Technologies and Advancements in MRS

Magnetic resonance spectroscopy (MRS) is a powerful tool in medical diagnostics, offering non-invasive insights into the metabolic profile of tissues. Emerging technologies and advancements in MRS are crucial for enhancing its capabilities and expanding its applications. One notable development area is the integration of artificial intelligence (AI) and machine learning (ML) into MRS. These technologies can significantly improve data analysis, enhance image processing, and automate diagnostic workflows, leading to more accurate and efficient diagnoses, particularly in complex cases. Additionally, quantum computing holds the potential to accelerate MRS data processing and analysis, enabling faster and more precise identification of metabolites [9]. Another key area of development is the utilization of cloud computing platforms. Cloud-based systems facilitate data storage, processing, and sharing, making MRS more accessible and scalable. This can also enable real-time collaboration among researchers and clinicians, thereby enhancing the utility of MRS in clinical settings. Furthermore, big data analytics can uncover patterns and correlations that may not be evident from individual studies, leading to a more comprehensive understanding of disease processes. Automation and robotics can also improve the accuracy and reproducibility of MRS measurements, streamlining workflows and reducing the risk of human error [40]. Future directions for MRS research include standardizing data acquisition and analysis protocols, integrating MRS with other imaging modalities such as MRI and PET, and exploring its potential in personalized medicine. Large-scale clinical trials and validation studies will be essential to establish the clinical utility of MRS across various applications, including cancer diagnosis and monitoring. Additionally, developing educational programs and training initiatives for healthcare professionals will be necessary to ensure the widespread adoption and effective use of MRS in clinical practice. The continued development and integration of emerging technologies into MRS will enable more accurate, efficient, and personalized diagnostic and therapeutic strategies [41].

Potential for Combining MRS With Other Imaging Techniques

The potential for combining magnetic resonance spectroscopy (MRS) with other imaging techniques is substantial. MRS offers metabolic insights that can complement the anatomical information MRI provides, thereby enhancing tumor characterization, staging, and treatment monitoring. Studies have demonstrated that integrating MRS with T2-weighted MRI improves tumor localization, volume estimation, and identification of recurrent disease after therapy. This synergy enhances MRI’s diagnostic accuracy by providing additional metabolic data that aids in distinguishing between benign and malignant lesions [21]. Parallel imaging techniques, such as sensitivity encoding (SENSE) and generalized autocalibrating partially parallel acquisitions (GRAPPA), can accelerate MRS acquisition by encoding spatial information through multiple receiver coils, significantly reducing scan times. Fast MRS techniques like echo-planar spectroscopic imaging (EPSI) can reduce scan times to one to two minutes, enabling rapid whole-brain spectroscopic mapping. This expedited acquisition capability enhances the feasibility of MRS for clinical use, particularly in urgent diagnostic and treatment scenarios [42]. Furthermore, correlating MRS-derived metabolic biomarkers, such as choline, with molecular and genetic features of tumors can provide a more comprehensive understanding of disease biology and guide personalized treatment strategies. This approach can help identify patients more likely to benefit from specific therapies, allowing for more targeted and effective treatment. Combining MRS with other imaging modalities and molecular/genetic biomarkers can improve diagnostic accuracy and clinical utility in evaluating brain tumors and other cancers. However, additional research is necessary to fully validate these multimodal approaches and their integration into clinical practice [43].

Limitations of the study

Despite the comprehensive approach, several limitations of this review must be noted. First, the literature search was restricted to English-language articles, which may introduce language bias and exclude relevant studies. Second, while key databases were used, some relevant studies might not be indexed, potentially leading to an incomplete representation of research. Third, the included studies vary in design, sample size, and methodology, leading to heterogeneity and inconsistent results. Fourth, the review includes studies of varying quality, which could affect the reliability of conclusions. Fifth, data extraction and interpretation are subject to human error and bias. Sixth, the field of MRS is rapidly evolving, and the review may not reflect the latest advancements. Seventh, there are limited clinical studies specifically on MRS for choline evaluation in gallbladder carcinoma, limiting the generalizability of findings. Finally, the focus on choline as a biomarker may overlook other important metabolic markers detected by MRS. Addressing these limitations in future research can provide a more robust assessment of MRS's role in evaluating choline levels in gallbladder carcinoma.

## Conclusions

In conclusion, magnetic resonance spectroscopy (MRS) holds promise as a valuable adjunctive tool in evaluating choline levels and other metabolic markers in gallbladder carcinoma (GBC). Through its ability to non-invasively assess biochemical changes within tissues, MRS offers clinicians insights into the altered cellular metabolism characteristic of GBC, potentially aiding in early detection, accurate staging, and treatment monitoring. Despite its advantages, challenges such as standardization of protocols, availability of specialized equipment, and interpretation of complex spectra remain significant. Future research efforts should focus on overcoming these challenges while exploring novel applications and advancements in MRS technology. Ultimately, integrating MRS into routine clinical practice can improve outcomes for patients with GBC by facilitating personalized treatment approaches and enhancing overall management strategies.
